# Hyper-Reactive Malarial Splenomegaly Syndrome (HMSS)

**DOI:** 10.7759/cureus.72

**Published:** 2012-11-30

**Authors:** Erwa Eltayib Elmakki

**Affiliations:** 1 Department of Internal Medicine, Faculty of Medicine/ Jazan University, Jazan, SAU

**Keywords:** lymphocytosis, malaria, high igm, gastroenterology, tropical splenomegaly syndrome (tss), hyper-reactive malarial splenomegaly syndrome (hmss)

## Abstract

Hyper-reactive malarial splenomegaly syndrome (HMSS) is a massive enlargement of the spleen due to an exaggerated immune response to repeated attacks of malaria.  Tropical splenomegaly syndrome (TSS) is the most frequent cause of massive tropical splenomegaly in malarious areas [1-2].  It is seen more commonly among residents of endemic areas of malaria.  It occurs mainly in tropical Africa, but also in parts of Vietnam, New Guinea, India, Srilanka, Thailand, Indonesia, South America, and the Middle East. TSS is characterized by massive splenomegaly, hepatomegaly, marked elevations in levels of serum IgM, and malaria antibody.

## Introduction and background

**Figure 1:**
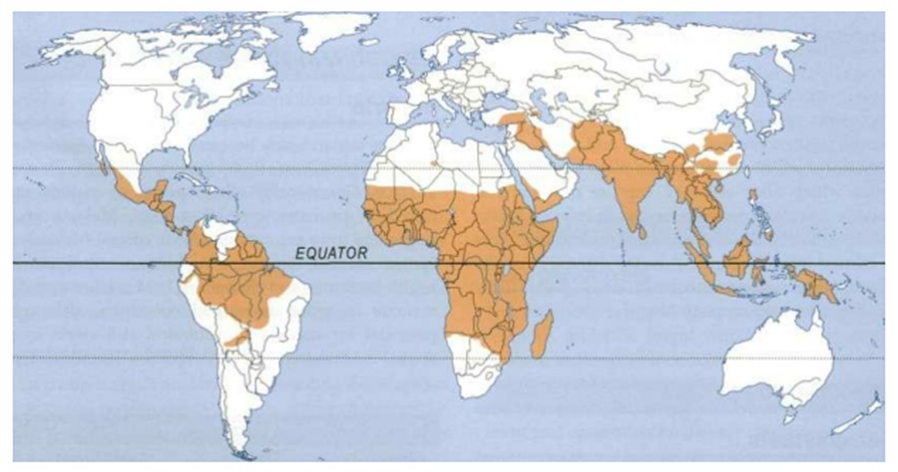
Geographical distribution of Malaria

Hyper-reactive malarial splenomegaly syndrome (HMSS) is a massive enlargement of the spleen due to an exaggerated immune response to repeated attacks of malaria. TSS is the most frequent cause of massive tropical splenomegaly in malarious areas [1-2]. It is seen more commonly among residents of endemic areas of malaria. It occurs mainly in tropical Africa, but also in parts of Vietnam, New Guinea, India, Srilanka, Thailand, Indonesia, South America, and the Middle East (Figure 1).

TSS is characterized by massive splenomegaly, hepatomegaly, and marked elevations in levels of serum IgM and malaria antibody. Hepatic sinusoidal lymphocytosis is also seen. In about 10% of African patients, it may be associated with peripheral lymphocytosis (B cells) [3]. TSS is more common in females than in males, with a female-to-male rate of 2:1. However, one study from Sudan revealed men to have a higher incidence [4]. TSS is most common in young and middle-aged adults, although the condition probably commences during childhood. TSS is rare in children less than eight years old but has been documented in three-year-old children [5].

## Review

A case report

A 32-year-old African male presented with a six-month history of left-sided abdominal mass which gradually increased in size, associated with dragging pain in the same area. He also had loss of weight and anorexia, but no fever, cough, night sweats, vomiting, or diarrhea. Past medical history included recurrent attacks of fever that usually responded to (over-the-counter) anti-malarial medications but was otherwise unremarkable.  His physical examination revealed normal vital signs, pallor and hepato-splenomegaly, spleen extended to the umbilicus, no ascites, lymphadenopathy, or signs of chronic liver disease. Laboratory tests showed: Hb: 9.5g/dl, WBC: 2800/cmm, platelets: 95,000/cmm, normochromic normocytic anemia, normal liver and renal function tests, negative blood film for malaria (repeated twice), positive anti-plasmodium falciparum antibodies, high serum IgM level (200 mg/dl), normal serum IgG, negative HIV serology, negative mantoux test, and normocellular bone marrow. Abdominal ultrasonography reported huge splenomegaly, plus hepatomegaly, no cirrhotic features, intra-abdominal lymphadenopathy, ascites, or evidence of portal hypertension. Chest x-ray was normal. The patient was put on proguanil, 200 mg daily; three month later, his condition started to improve in terms of subsidence of symptoms, correction of hematological disturbances and regression of splenic size; he was advised to have life-long anti-malarial treatment.

Pathogenesis

Studies on the pathogenesis of HMSS suggest a critical role of aberrant immunologic response to malaria antigens after repeated infection, resulting in splenic hypertrophy, sometimes associated with secondary hypersplenism [6].

The interaction between repeated malarial infection and genetic factors class II HLA DR2, IGHG3G (Igg-3 chain C region) and enviromental factors lead to the production of cytotoxic IgM antisuppressor lymphocyte (CD8+) antibodies. This results in inhibition of suppressor T-cells. T-cells are the regulator IgM production. This ends up with uninhibited B-cell formation of IgM and cryoglobulins (IgM aggregates and immune complexes). The need to clear these macromolecular aggregates stimulates the reliculoendothelial system, leading to hyperplasia. This causes the progressive and massive enlargement of the spleen and liver. The spleen is greatly enlarged and shows dilated sinusoids lined with reticulum cells with marked erythrophagocytosis and lymphocytic infiltration of the pulp. The liver shows sinusoidal dilatation, infiltration with lymphocytes, and hyperplasia of the Kupffer's cells with phagocytosis of cellular debris and red cells [7].

Clinical presentation

**Figure 2:**
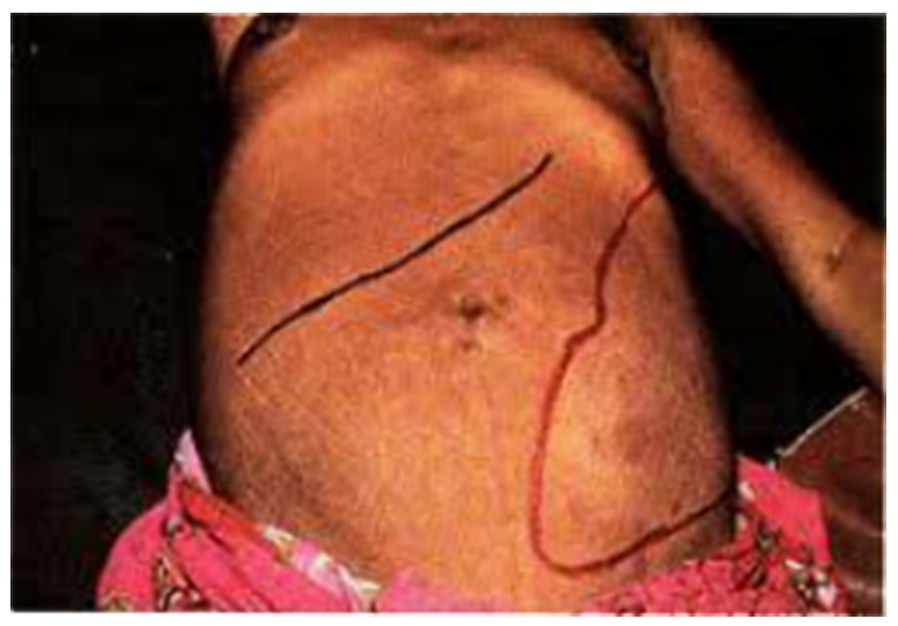
Massive Splenomegaly

The most common presenting symptoms of TSS are chronic abdominal swelling (64%) and dragging abdominal pain (52%), mainly during adult life [8]. Almost all patients (97%) have weight loss. Bleeding complications, such as epistaxis, is uncommon because thrombocytopenia secondary to hypersplenism is usually mild [9]. Some patients may experience recurrent sharp pains in the upper abdomen, possibly due to perisplenitis or splenic infarcts. Other patients may have weight loss and cachexia. On examination, there is massive splenomegaly and hepatomegaly (Figure 2). The patients typically lack malarial parasitaemia and fever on presentation [10].

Diagnosis

For diagnosing TSS, other causes of massive splenomegaly need to be excluded, such as visceral leishmaniasis (kal-azar), schistosomiasis (portal hypertension), myelofibrosis, and chronic myeloid leukemia (CML) (Table 1).

**Table 1:** Differential Diagnosis of Massive Splenomegaly

Visceral Leishmaniasis
HMSS
Portal Hypertension
Schistosomiasis
Myeloproliferative diseases
eg: Chronic myeloid leukemia (CML), polycythemia vera, essential thrombocythemia
Lymphoproliferative disorders eg: lymphoma and chronic lymphatic leukaemia (CLL).
Idiopathic non-tropical splenomegaly
Spleen cysts or tumours
Gaucher disease
Thalassemia major

However, exclusion of other disease processes causing splenomegaly is not enough to establish a diagnosis of hyper-reactive malarial syndrome (HMS). Fakunle was the first one who described diagnostic criteria for the definitive diagnosis of HMS. Bates and Bedu-Addo modified these major criteria in 1997 [11-12] (Table 2).

**Table 2:** Diagnostic criteria for HMSS

Major criteria include the following:
Gross splenomegaly 10 cm or more below the costal margin in adults for which no other cause can be found
Elevated serum IgM level 2 standard deviations or more above the local mean
Clinical and immunologic responses to antimalarial therapy
Regression of splenomegaly by 40% by 6 months after start of therapy
High antibody levels of Plasmodium species (≥ 1:800)
Minor criteria include the following:
Hepatic sinusoidal lymphocytosis
Normal cellular and humoral responses to antigenic challenge, including a normal phytohemagglutination response
Hypersplenism
Lymphocytic proliferation
Familial occurrence

Laboratory features

In TSS, the peripheral smear shows normocytic normochromic anaemia with elevated reticulocyte count. Pancytopenia may also be seen as a result of hypersplenism. Malarial parasites are not found in the peripheral blood. There is elevation in the serum levels of polyclonal IgM with cryoglobulinaemia, reduced C_3_ and the rheumatoid factor may be positive. Increased levels of IgM and antimalarial antibody, hepatic sinusoidal lymphocytosis on liver biopsy, and response to antimalarial therapy (improvement in clinical condition as well as reduction in IgM and malarial antibody titre within three months of continuous antimalarial treatment) favour a diagnosis of tropical splenomegaly syndrome [13].

**Figure 3:**
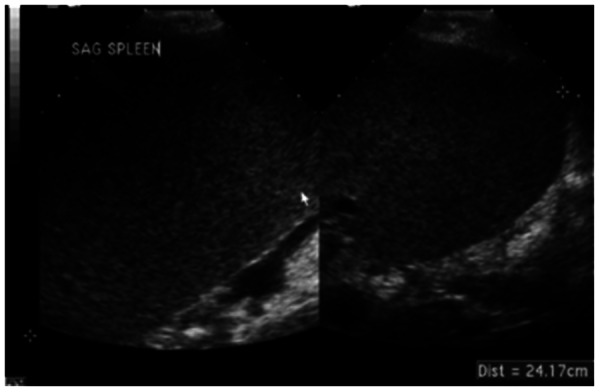
Ultrasound image of massive splenomegaly

Treatment

Antimalarials are the cornerstones of treatment of HMSS. The selection of drug is based on the pattern and prevalence of drug resistance in the patient's geographic area. In malaria endemic areas, treatment should be prolonged (months to years) and continued regularly. However, the exact duration of treatment has not been ensured. Response may be seen within months after commencing treatment, and relapses may occur when therapy is discontinued [14].

Antimalarials clear the antigenic stimulus caused by repeated malarial infections and helps the immune system to return to normal. The selection of antimalarial depends upon the local sensitivity pattern. Chloroquine weekly or Proguanil daily have been found to be effective. Pyrimethamine may be an alternative to the above medications [15]. Data regarding the usefulness of other antimalarial drugs in HMSS is limited. The response to therapy is guided by reduction in splenic size, a decrease in serum IgM levels, correction of anemia and other blood dyscriasis, and general improvement in the patient's well-being.

Severe anaemia may require blood transfusion. Bearing in mind the risks of splenectomy, it may be useful in only those with splenic lymphoma. Splenic irradiation or antimitotic therapy are not of benefits and may be even dangerous [16].

## Conclusions

Take home message

1) HMSS results from abnormal immunological response due to repeated attacks of malaria and is usually seen in those who live in malaria endemic areas.

2) The main features of this syndrome are hepato-splenomegaly, high IGM levels, and hepatic lymphocytosis on liver biopsy, in addition to features of hypersplensim.

3) HMSS should usually be included in the differential diagnosis of massive splenomgaly, especially in tropical and subtropical countries.

4) Lifelong anti-malarials are the mainstay of treatment of HMSS.

## References

[1] Fakunle YM (1981). Tropical splenomegaly. Part 1: Tropical Africa. Clin Haematol.

[2] McGilvray ID, Serghides L, Kapus A, Rotstein OD, Kain KC (2000). Nonopsonic monocyte/macrophage phagocytosis of Plasmodium falciparum-parasitized erythrocytes: a role for CD36 in malarial clearance. Blood.

[3] Weather DJ, Ledingham JGG, Warrell DA (1996). Tropical splenomegaly syndrome. Oxford Text Book of Medicine.

[4] Allam MM, Alkadarou TA, Ahmed BG (2008). Hyper-reactive Malarial Splenomegaly (HMS) in malaria endemic area in Eastern Sudan. Acta Trop.

[5] Verma S, Aggarwal A (2007). Hyper-reactive malarial splenomegaly: rare cause of pyrexia of unknown origin. Indian J Pediatr.

[6] Greenwood BM, Fakunle YM (1979). The tropical splenomegaly syndrome: a review of its pathogenesis. The role of the spleen in the immunology of parasitic diseases.

[7] Fakunle YM (1981). Tropical splenomegaly. Part 1: Tropical Africa. Clin Haematol.

[8] Mitjà O, Hays R, Malken J, Ipai A, Kangapu S, Robson J (2011). HMS-related hemolysis after acute attacks of Plasmodium vivax malaria. Am J Trop Med Hyg.

[9] Grane GG (1986). Hyperreactive malarious splenomegaly "Tropical splenomegaly syndrome". Parasitol Today.

[10] Fakunle YM (1981). Tropical splenomegaly. Part 1: Tropical Africa. Clin Haematol.

[11] Bates I, Bedu-Addo G (1997). Review of diagnostic criteria of hyper-reactive malarial splenomegaly. Lancet.

[12] Hoffman SL, Piessens WF, Ratiwayonto S (1984). Reduction of suppressor T lymphocytes in the tropical splenomegaly syndrome. N Engl J Med.

[13] Van den Ende J, van Gompel A, van den Enden E (2000). Hyperreactive malaria in expatriates returning from sub-Saharan Africa. Trop Med Int Health.

[14] Manenti F, Porta E, Esposito R, Antinori S (1994). Treatment of hyperreactive malarial splenomegaly syndrome. Lancet.

[15] Sagoe AS (1970). Tropical splenomegaly syndrome: Long-term proguanil therapy correlated with spleen size, serum IgM, and lymphocyte transformation. BMJ.

[16] Crane GG (1986). Hyperreactive malarious splenomegaly (tropical splenomegaly syndrome). Parasitol Today.

